# Lack of Toxic Interaction between Fusariotoxins in Broiler Chickens Fed throughout Their Life at the Highest Level Tolerated in the European Union

**DOI:** 10.3390/toxins11080455

**Published:** 2019-08-02

**Authors:** Jean-Paul Metayer, Angelique Travel, Amandine Mika, Jean-Denis Bailly, Didier Cleva, Cyril Boissieu, Jean Le Guennec, Pascal Froment, Olivier Albaric, Sophie Labrut, Gurvan Lepivert, Eric Marengue, Didier Tardieu, Philippe Guerre

**Affiliations:** 1 ARVALIS-Institut du Végétal, Station expérimentale, 91720 Boigneville, France; 2 ITAVI, Centre INRA Val de Loire, 37380 Nouzilly, France; 3 Université de Toulouse, INP, ENVT, Equipe Biosynthèse et Toxicité des Mycotoxines, UMR Toxalim, F-31076 Toulouse, France; 4 Chêne Vert Conseil, Z Bellevue II–35220 Chateaubourg, France; 5 Finalab, 4 bis rue Th. Botrel, BP 351, 22603 Loudéac CEDEX, France; 6 Team Sensor, UMR 7247 INRA/CNRS/Université de Tours/IFCE 37380 Nouzilly, France; 7 ONIRIS, Site de la Chantrerie, BP 40706, 44307 Nantes CÉDEX 3, France; 8 LABOCEA, 7 rue du Sabot, CS 30054, Zoopole, 22440 Ploufragan, France; 9 Université de Toulouse, INP, ENVT, UR Mycotoxicologie, F-31076 Toulouse, France

**Keywords:** feed, broilers, deoxynivalenol, fumonisins, zearalenone, interactions

## Abstract

*Fusarium* mycotoxins (FUS) occur frequently in poultry diets, and regulatory limits are laid down in several countries. However, the limits were established for exposure to a single mycotoxin, whereas multiple contamination is more realistic, and different studies have demonstrated that it is not possible to predict interactions between mycotoxins. The purpose of this study was thus to compare the toxic effect of deoxynivalenol (DON), fumonisins (FB) and zearalenone (ZON), alone and in combination on broiler chickens, at the maximum tolerated level established by the EU for poultry feed. Experimental corn-soybean diets incorporated ground cultured toxigenic *Fusarium* strains. One feed was formulated for chickens 0 to 10 days old and another for chickens 11 to 35 days old. The control diets were mycotoxin free, the DON diets contained 5 mg DON/kg, the FB diet contained 20 mg FB1 + FB2/kg, and the ZON diet contained 0.5 mg ZON/kg. The DONFBZON diet contained 5, 20, and 0.5 mg/kg of DON, FB1 + FB2, and ZON, respectively. Diets were distributed *ad libitum* to 70 broilers (male Ross PM3) separated into five groups of 14 chickens each reared in individual cages from one to 35 days of age. On day 35, after a starvation period of 8 h, a blood sample was collected, and all the animals were killed and autopsied. No difference between groups that could be attributed to FUS was observed in performances, the relative weight of organs, biochemistry, histopathology, intestinal morphometry, variables of oxidative damage, and markers of testicle toxicity. A significant increase in sphinganine and in the sphinganine to sphingosine ratio was observed in broilers fed FB. Taken together, these results suggest that the regulatory guidelines established for single contamination of broiler chickens fed with DON, FB, and ZON can also be used in the case of multiple contamination with these toxins.

## 1. Introduction

The worldwide contamination of raw materials with mycotoxins is a safety concern in both humans and in animal species, including poultry. In Europe, the occurrence of aflatoxins and ochratoxins is relatively low. The co-occurrence of mycotoxins produced by *Fusarium* (Fusariotoxins, FUS) in avian feed is the most frequent [1,2,3,4]. Among FUS, four compounds are subject to international and European guidelines on their maximum tolerated levels in avian feed. These toxins are members of group A and B trichothecenes, T-2 toxin (T-2) and deoxynivalenol (DON) respectively, the myco-estrogenic zearalenone (ZON), and fumonisins B1 and B2 (FB1 and FB2), belonging to group B of fumonisins (FB). Among the FUS with regulatory limits, DON, FB and ZON occur the most frequently in poultry diets, whereas T-2 is less frequent in Europe [2,5,6].

Although the effects of mycotoxins on health have been intensively studied in different poultry species, most information available on toxicity was obtained using exposure to a single mycotoxin, and most studies involved a high level of exposure. By contrast, combined effects of low levels/doses of toxins are more likely but less well understood [7,8,9,10,11]. Most studies on the combined effects of mycotoxins were done using aflatoxin B1 in association with FUS [12,13,14,15,16,17]. Only very few studies have investigated the combined effects of FUS in poultry. One trial investigated the effects of high doses of FB1 and DON on broiler chicks from hatching up to 21 days of age [18]. In this assay, 300 mg FB1/kg feed combined with 15 mg DON/kg feed, had some synergic toxic effects on serum biochemistry (increases in the activity of aspartate aminotransferase, lactate dehydrogenase, and gamma glutamyl transferase), and a less than additive effect on body weight gain (BWG), whereas an antagonistic effect was observed on the relative weight (RW) of the heart. Another study, also using FB1 and DON, was conducted on chickens exposed to a subclinical dose of *Eimeria* spp., responsible for coccidiosis [19]. The conclusion of that study was that interactions between DON and FB1 depended to a large extent on the endpoint assessed, with three endpoints reporting antagonism, nine additivity, and two synergism. Another trial investigated the combined effects of FB and ZON on nutrient digestibility in female broilers [20]. In this assay, the effect of ZON (0 vs. 1 mg/kg feed) in the presence of different concentrations of FB (3.15 and 5.5 mg/kg feed) was investigated. The main result was that nutrient digestibility may be improved by dietary ZON at a level of 1 mg/kg but was not affected by different levels of FB.

Taken together, these results suggest that it is not possible to predict interactions between FUS, as already reported for all other mycotoxins [8,21]. Complementary data were thus required to draw conclusions concerning the maximum tolerated doses established for single contamination in the case of multiple contaminations. The purpose of this study was to compare the toxic effects of DON, FB, and ZON, alone and in combination, at the maximum tolerated level defined for poultry feed by the EU [22]. The study was conducted on broiler chicks from hatching until 35 days of age to maximize the animal’s exposure to the toxins. We investigated the effects on performance, organ weight, and histopathology, these being the parameters most frequently used to reveal deleterious effects, but also on some more specific markers of toxicity such as intestinal morphometry and the number of goblet cells, oxidative damage, sphingolipid metabolism and male reproductive toxicity.

## 2. Results

### 2.1. Diets and Performances

Table 1 shows the expected and measured levels of FUS in the diets. Although traces of DON, FB, and ZON were found in the control diets, their levels were always <100 µg/kg and were considered not significant. Measured concentrations of DON, FB, and ZON in the contaminated diets differed between 10% and 20% from the expected levels. The biggest difference was found for DON in the DONFBZON diet. In all the diets, no unexpected FUS, other than DON, FB, and ZON, were detected or were <20 µg/kg. All other mycotoxins were below the limit of quantitation.

Neither mortality nor any signs of mycotoxicosis were observed in any of the chickens during the 35-days study. The effects of feeding FUS on performances at the two ages are detailed in Table 2. From 0 to 10 days, no differences among groups were observed in body weight (BW), feed consumption (FC), daily weight gain (DWG), and feed conversion ratio (FCR). By contrast, a significant increase in FC was observed in chickens fed the ZON and DONFBZON diets from 11 to 35 days, but had no consequence for BW and FCR. Over the entire period (0 to 35 days), the only significant difference observed among the groups was in FC, but no group differed significantly from the control group (data not shown).

### 2.2. Relative Organ Weights and Histopathology

Although some signs of runny noses, puddle hearts, and signs of reactivity in the caeca tonsils were found in some chickens, these signs were not more marked in one group than in another. The effects of FUS on the relative weights (RW) of liver, kidney, intestine, caecum, gizzard, and hearth are shown in Table 2. Although some differences in the RW of the liver were found among groups, no group differed from the control group. No significant difference in the RW of pancreas, spleen, proventriculus, duodenum, jejunum, ileum, caeca tonsils, and Fabricius bursa was observed among groups (Table 2). The color of the liver and caeca tonsils did not differ among groups.

Histopathological examination of the hepatic and renal samples revealed discrete non-specific inflammatory lesions with no difference in frequency or intensity among groups. Lymphoid hyperplasia with signs of typhlitis was observed in the caeca mucosa. These lesions varied greatly depending on the section examined and were not more frequent in one group than in another. Characterization of intestinal morphometry of duodenum, jejunum, and ileum revealed no significant differences in the size of the villus and crypts in the duodenum and ileum among groups whereas a small increase in crypt depth of the jejunum was observed in broilers fed the diet containing FUS (Table 3). Moreover, no significant difference in the surface area and the villus to crypt ratio of the duodenum and ileum was found among groups (data not shown). By contrast, in the jejunum, the surface area of the crypt increased in broilers fed FUS whereas the villus to crypt ratio decreased and the surface area of the villus was not affected (Table 3). No significant difference in the number of goblet cells in the duodenum, jejunum, and ileum was found among groups.

### 2.3. Serum Biochemistry and Hematology

The effect of FUS on biochemistry and hematology are presented in Table 4. No statistical difference in proteins, cholesterol, IgA and Hb concentrations was found among groups. In the same way, no effect was observed on LDH, ALP, and ALT activities or on the number of erythrocytes and leucocytes. Only a small but significant increase in the concentration of uric acid was observed in chickens fed with ZON alone.

### 2.4. Oxidative Markers and Antioxidant Enzyme Activity in Plasma and Liver

Different variables were investigated to identify oxidative damage to broiler chickens (Table 5). MDA and TGlu contents in plasma, and MDA, TGlu, and GSSG contents in the liver remained unchanged after exposure to FUS. The GSH/GSSG ratio in the liver was also unaffected by the toxins (data not shown). Investigation of the activities of most of the enzymes involved in defense against oxidative damage, SOD, CAT, GsPx, and GsRed, both in plasma and liver, failed to reveal any effect of the FUS, alone or in association.

### 2.5. Testis Toxicity

The weight of the testis was similar in all the groups but the ZON group presented a 25% increase in weight compared to the control group (Table 6). The diameter of the seminiferous tubule in the ZON group was also 17% larger than in the controls, whereas a 21% decrease was observed in the DONFBZON group. Cell death observed by ISEL staining failed to reveal any significant difference among groups (data not shown). The activity of the cleaved caspase 3 was similar in the five groups (Table 6).

**Figure 1:**
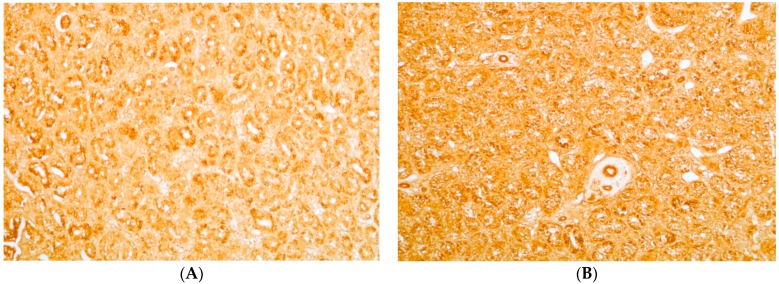

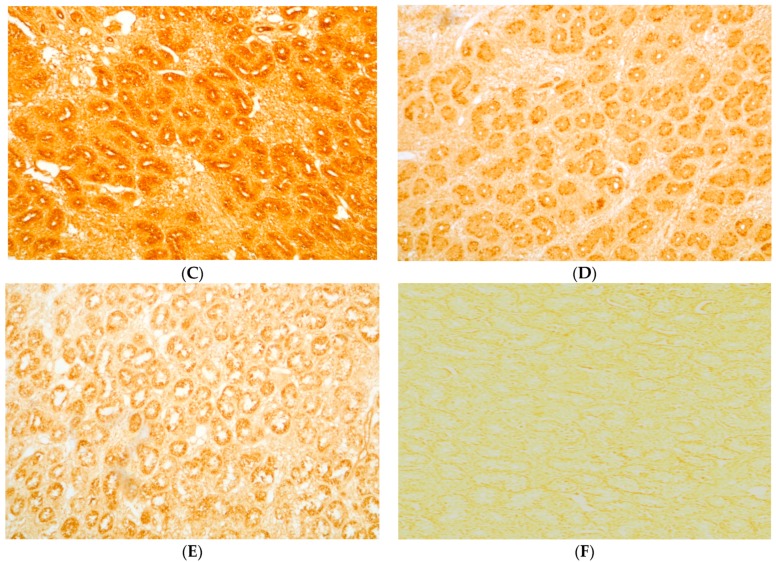
Immunohistochemistry detection of germ cells in testis with primary antibody against chicken Vasa (**A**–**E**) or rabbit IgG (**F**). Magnification ×20. Testes were obtained from chickens fed experimental diets with a final concentration of 5 mg DON/kg diet (**B**), 20 mg FB1 + FB2/kg diet (**C**), 0.5 mg ZON/kg diet (**D**) and 5 mg DON + 20 mg FB1 + FB2 + 0.5 mg ZON/kg diet (**E**). The control diet (**A**,**F**) was free of mycotoxins.

As revealed by VASA staining, germ cells were present in the testis (Figure 1). The number of immature germ cells estimated by intra-testis Dazl content was higher in the FB group than in the other groups. This increase was associated with higher PCNA content, a marker of cell proliferation, in the FB group.

Inflammation markers (IFN-γ and IL1β) and oxidative stress measured by TAC and catalase activity were not affected by exposure to mycotoxins. Despite the fact that testosterone and cAMP production was measured in an immature testis (before puberty, 35 days post-hatching), no effect was observed.

### 2.6. Sphinganine to Sphingosine Ratio

**Figure 2:**
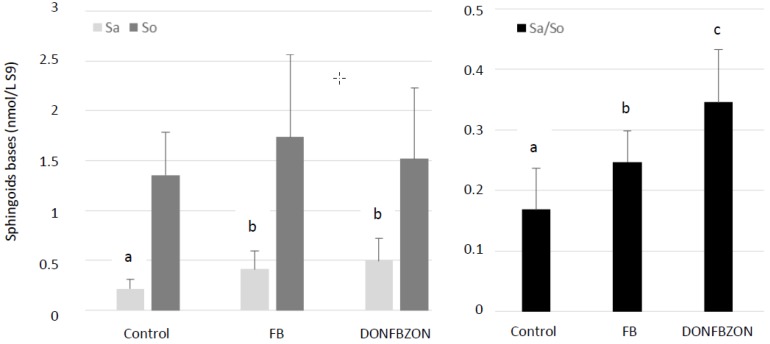
Effects of fumonisins on liver sphinganine (Sa). sphingosine (So) and on the sphinganine to sphingosine ratio (Sa/So) in broiler chickens fed for 35 days with a diet containing 20 mg FB1 + FB2/kg (FB) or a diet containing deoxynivalenol, FB1 + FB2, and zearalenone (DONFBZON) at respective concentrations of 5, 20 et 0.5 mg/kg. The control diet (Control) was free of mycotoxins. When ANOVA revealed a significant difference (*p* < 0.05) between groups, means were compared (Kruskall–Wallis). Different letters identify statistically different groups (*p* < 0.05).

Free Sa and So were quantified in the liver and the Sa to So ratio was calculated (Figure 2). No significant difference in So concentration was observed among groups whereas the Sa level and the Sa to So ratio increased in broilers fed with the FB and DONFBZON diets (ANOVA, *p* < 0.05). Complementary comparison of means (Kruskall–Wallis) revealed no significant difference in the Sa level in the liver between broilers fed the FB and DONFBZON diets, whereas the Sa/So ratio was significantly higher in chicken fed the DONFBZON diet than in chickens fed the FB diet (*p* < 0.05). This result was surprising because the total amount of FB1 + FB2 was only slightly higher in the FB diet than in the DONFBZON diet, 23.13 and 19.23 mg FB1 + FB2/kg, respectively (Table 1). No significant difference in Sa and So contents or in the Sa/So ratio in the liver was observed between chickens fed the control diet and chickens fed the DON and ZON diets (data not shown).

## 3. Discussion

In this study, a single exposure to DON, ZON, and FB from 0 to 35 days did not lead to any alteration in the performances of the broiler chickens or have a significant effect on organ weight, histopathology or biochemistry. These results are not surprising because single exposure involved FUS concentrations in diets that are equal to or below regulatory limits in poultry diets and these species are known to tolerate FUS [2,11,23,24,25]. Moreover, only a weak effect of FUS was found on intestinal morphometry of duodenum, jejunum, and on the number of goblet cells, in agreement with a long term study conducted in slow-growing chickens fed 2 to 10 mg DON/kg diet [26]. Conversely, some studies reported that DON altered the small intestinal morphology and the length of the villi in the jejunum in broilers [27,28] and that FB reduced the length of the villi and the crypt depth of the ileum [29]. Differences between studies could be due to the way the toxins were added to the diet or to external factors. Indeed, studies carried out to evaluate the effects of mycotoxins can be conducted by naturally contaminated feedstuffs, culture material or pure crystalline toxins. The response from the different sources is an important contributor to the susceptibility in the model (i.e., naturally contaminated material is often more toxic in research environments presumably because other lesser-known or unknown compounds may contribute to their effects). In this study, the objective was to compare single- and multiple-dose effects of DON, ZON, and FB with the maximum recommended in poultry feed in Europe. The use of mycotoxins obtained from fungal culture extracts appeared the best way to obtain contaminated feeds with the desired exposure scenarios. Indeed, the culture material, although partially purified, still contains the metabolites that are formed in the synthesis of the final mycotoxin. Moreover, it is reasonable to assume that the mix of the extract with the feed during its manufacture results in little variation in mycotoxins bioavailability, at least compared to the administration of toxins by oral gavage [30]. With regard to external factors, this study was carried out under conditions aimed at optimizing the measurement of the effects of toxins on healthy animals. For this reason, breeding in individual cages was preferred to breeding in parquet floors. In general, farming management can change the performance and response of animals to various factors, including stress. Similarly, concomitant exposure to infectious or parasitic agents may alter the response of animals to mycotoxins, as observed in broiler chickens fed DON and FB at a not toxic level that amplified the severity of coccidiosis [19].

Only a few investigations have been conducted on oxidative stress in the course of FUS exposure in broiler chickens. One study reported that 7.54 mg DON/kg feed down-regulated heme-oxygenase and upregulated xanthine oxidoreductase mRNA in the liver [31]. Another study reported that 100 mg FB1/kg of feed increased hepatic MDA levels and CAT activity in chickens [32]. Moreover, the intake of contaminated feed containing DON and ZON in combination was reported to significantly reduce the activity of glutathione peroxidase and to increase the level of MDA in liver tissue [33]. In the present study, several variables were measured in plasma and liver to reveal oxidative stress, but none was significantly changed by FUS exposure.

Effects of FUS on reproductive function are usually not reported in avian species, except with very high levels of ZON [11,34]. The weights of testes were significantly reduced in broilers fed 200 to 400 mg ZON/kg [35]. In mammals, the most toxic effect of FUS other than ZON on reproductive function has been mild-to-moderate lesions of testes with Sertoli cell degeneration and impaired spermatogenesis observed in rabbits fed 0.13 to 5 mg FB1/kg diet for 175 days [36,37]. Studies conducted in mice suggested that DON may have an adverse effect on the epididymal weight at 10 mg/kg of feed for 90 days with slight changes in relative testis weight and spermatid counts, but no histological changes [38]. In the present study, no significant difference was observed in the variables measured to reveal toxicity of FUS in broiler testes except a decrease in the diameter of the seminiferous tubule and a decrease in catalase activity in the DONFBZON group that are difficult to interpret. All these results confirm that broilers are less sensitive to the toxic reproductive effects of FUS than mammals, even when a mixture of toxins was used.

Using biomarkers of effects is a good way to reveal the effect of mycotoxins on health at a level of exposure lower than one that is toxic. Alteration of sphingolipid metabolism has been known for several years to be the best biomarker of FB exposure in most animal species, including poultry [9,11]. In the present study, a significant increase in the level of Sa in liver concomitant with an increase in the Sa/So ratio was observed in the FB-treated group compared to the control group. This result is in agreement with previous data obtained with higher levels of FB in broiler chicken diets, and with data obtained in plasma using the same dose [29,38]. Together, the lack of histopathological damage to the liver and the lack of increase in LDH activity in plasma confirmed the alteration of sphingolipid metabolism occurred at a level of FB that is not hepatotoxic in broiler chickens.

Taken together, these results confirm that single exposure of broiler chickens from 1 to 35 days of age to FUS concentrations in diets that are equal to or below regulatory limits for poultry feed had no deleterious effect on health. Interestingly, no additive, synergistic or antagonist effect on the variables measured was observed in broilers exposed to the diet containing a mixture of DON, FB, and ZON at similar levels as those used in formulation trials conducted with a single toxin. Although previous studies on chickens revealed interactions between FUS, no study was conducted using EU regulatory limits. Concerning sphingolipids in the liver, the Sa/So ratio was significantly higher in chickens fed the DONFBZON diet than in chickens fed the diet containing FB alone, whereas the measured concentration of FB1 + FB2 was higher in the FB diet than in the DONFBZON diet. The apparent synergistic effect of FUS on the Sa/So ratio should be interpreted with caution and should take the effects on Sa and So and not only the effects on the Sa/So ratio into account. Indeed, previous studies on ducks and turkeys with low doses of FB demonstrated that the level of FB1 in the diet is always correlated with an increase in Sa content in the liver. By contrast, the effects of low doses of FB1 on So content are less pronounced and vary with the study [39,40,41]. Moreover, most studies on avian species have shown that toxic levels of FB in diets are responsible for the liberation of LDH by the hepatocytes in plasma and for an increase in LDH activity in this medium [41,42,43,44], which was not the case in our chickens fed the DONFBZON diet.

In conclusion, this study demonstrated for the first time the lack of strong interactions between DON, FB and ZON fed alone or in combination to broiler chickens from 1 to 35 days in age on the variables measured. Together, the lack of effect of these toxins on performances, organ weight, biochemistry, histopathology, variables of oxidative damage and markers of testis toxicity suggest that the regulatory guidelines established for single contamination of broiler chickens fed DON, FB and ZON can be used in the case of multiple contamination with these toxins.

## 4. Materials and Methods

### 4.1. Fusariotoxin Production and Experimental Diets

All the experimental diets containing mycotoxins incorporated ground cultured toxigenic *Fusarium* strains. Briefly, fumonisins were produced on crushed corn at 25 °C using *F*. *verticillioides* strain L12. Deoxynivalenol was produced by growing *F. graminearum* strain I159 on wheat at 23 °C, and zearalenone was obtained by culturing *F. graminearum* strain I171 on rice at 21 °C. After four weeks of growth, the cultured *Fusarium* species were dried at 90 °C for 3 h, ground and sieved through a 0.6 mm mesh. The concentrations of mycotoxins in the powders were measured by HPLC-MSMS.

Experimental corn-soybean diets were used to best meet the nutritional needs of the animals. One feed was formulated for 0 to 10 days of age (CP = 22%, ME = 2880 kcal/kg) and another for 11 to 35 days of age (CP = 19.5% and ME = 3050 kcal/kg). Powders containing the mycotoxins were incorporated to obtain 10 different experimental diets (Table 1). The control diets (Control) were free of mycotoxins. The deoxynivalenol (DON), fumonisin (FB), and zearalenone (ZON) diets were made by incorporating powdered cultured materials to obtain 5 mg DON/kg, 20 mg FB1 + FB2/kg, and 0.5 mg ZON/kg, respectively. The three toxins were added in the diet that contained the mixture of fusariotoxins (DONFBZON) at concentrations of 5, 20 and 0.5 mg/kg for DON, FB1 + FB2, and ZON, respectively. The concentrations of mycotoxins in the different diets was checked by HPLC-MSMS.

### 4.2. Mycotoxin Analysis

The concentrations of mycotoxins in the culture material and in the experimental diets were analysed by HPLC-MSMS according to the AFNOR V03-110 recommendation [45]. All reactants were of HPLC analytical grade except pure water and acetic acid that were of HPLC-MS grade (Fluka, Buchs, Switzerland). Stock solutions of standard mycotoxins (Romer Labs, 3131 Getzersdorf, Austria) prepared in acetonitrile were solubilized with 0.01% of acetic acid for HPLC-MS/MS calibration. Mycotoxins assayed were diacetoxyscirpenol, 15 monocetoxyscirpenol, T2 toxin, HT2 toxin, T2 tetraol, verrucarol, desoxynivalenol, desoxynivalenol-3-glucoside, deepoxy-deoxynivalenol, 15-acetyl-deoxynivalenol, 3-acetyl-deoxynivalenol, fusarenone x, nivalenol, roridin A, verrucarin A, fumonisin B1, fumonisin B2, fumonisin B3, moniliformine, zearalenone, alpha-zearalenol, beta-zearalenol, zearalenone, alpha-zearalanol, beta-zearalanol, tenuazonic acid, ergocornine, ergocristine, ergocryptine, ergometrine, ergosine, ergotamine, aflatoxin B1, aflatoxin B2, aflatoxin G1, aflatoxin G2, ochratoxin A, ochratoxin B, alpha-ochratoxin, cyclopiazonic acid, citrinin, patulin, and sterigmatocystin. The HPLC was performed using Hewlett Packard type 1100 (Hewlett Packard, Eybens 38, France) in the following conditions: Column 250 mm × 4.6 packed with C_18_ phase (VWR Pessac 33, France Applied Biosystems, Foster City, CA, USA). Mobile phase: Ammonium acetate 1nM and 0.0001% acetic acid/methanol and 1% acetonitrile. A linear gradient was applied for 40 min at a flow rate of 1 mL/min. Detection was performed with a quadrupole tandem mass spectrometer API 4000 (Applied Biosystems, Foster City, CA, USA) at a source temperature of 500 °C with a 4500 V ion spray voltage in positive and negative mode interface. Each mycotoxin was identified and quantified on two or three transitions. Experimental diets (1 kg) were ground to a fine powder and sifted through a 0.5 mm particle size filter. Five g of sieved samples were extracted for 2 h by reversal agitation with 20 mL of acetonitrile/water. The extract was centrifuged, and 3 mL of the aqueous phase were evaporated to dryness. The dry residue was dissolved in a solution of 0.01% acetic acid methanol (2/1, *v*/*v*), filtered on a syringe, and injected in the HPLC-MS/MS. The limit of quantitation ranged from 1 to 10 µg/kg, depending on the mycotoxin.

### 4.3. Animal Husbandry and Sample Collection

All experimental procedures with animals were in accordance with the French National Guidelines for the care and use of animals for research purposes. The experimental protocol was approved by the French Ministry of Higher Education and Research and registered under number 02032.01. Seventy broilers (male Ross PM3) were reared in individual cages in an experimental station (ARVALIS—Institut du vegetal, Villerable, France). Each experimental diet was distributed to 14 broilers ad libitum throughout the experiment along with ad libitum access to water. Individual body weight and feed consumption were measured weekly. On the 35th day of age, after a starvation period of 8 h, a blood sample was collected. Samples were centrifuged and serum was collected and stored at −80 °C until analysis. The animals were stunned by electrocution and killed by exsanguination. Autopsies were performed on all the animals to investigate macroscopic lesions and to examine the color of the liver and cecum tonsils. The heart, liver, spleen, thymus, pancreas, kidneys, and testicles were collected and weighed. The intestine was emptied, then the gizzard, proventriculus, duodenum, jejunum, ileum, and caeca (including caeca tonsils) were isolated and weighed. The length of the duodenum, jejunum and ileum segments was measured. The color of the liver and cecum tonsils was measured with a chromameter (Konica Minolta, Europe). Samples of liver, kidney, spleen, duodenum, jejunum, ileum, caecum, caeca tonsils, and Fabricius bursa were placed in 10% formaldehyde for microscopic examination. The remaining liver and testicles were stored at −80 °C until analysis.

### 4.4. Biochemistry, Hematology, and Histopathology

Plasma concentrations of lactate dehydrogenase (LDH) EC 1.1.1.27, alkaline phosphatase (ALP) EC 3.1.3.1, and alanine aminotransferase (ALT) EC 2.6.1.2 were analyzed using a clinical chemistry KONELAB 20 analyzer (Fisher Scientific SAS, Illkirch, France) and are expressed in UI/L according to international guidelines. Proteins, cholesterol, and uric acid were measured according to the manufacturer’s instructions and are expressed in g/L or mmol/L of plasma. Immunoglobulin A (IgA) assays were performed using the chicken IgA Elisa kit following the manufacturer’s instructions (Bethyl Laboratories Inc., Montgomery, TX, USA). Hemoglobin (Hb) and erythrocytes contents were measured using Hycel Celly analyzer (EUROCELL Diagnostics, Rennes, France) and are expressed in mg/L and in 10^3^ number of cells/mL, respectively. The white blood cell count was done manually in Malassez cells, and the results are expressed in 10^3^ number of cells/mL.

Except for the testes, fixed tissues were trimmed, embedded in paraffin, and 4 μm sections cut with a microtome, and stained with hematoxylin, eosin, and saffron (HES). All the tissues from the control and treated groups were examined microscopically. Fixed testes were processed in paraffin and serially sectioned at 7 µm. The diameter of the seminiferous tubules was measured with round or nearly round tubules from each testicular section stained with Meyer’s hematoxylin (Sigma, l’Isle d’Abeau Chesnes, France). At least 40 measurements of the diameter of the transverse sections of seminiferous tubules were measured using an ocular measuring device. Four different chickens were analyzed per treatment.

### 4.5. Immunohistochemistry and Detection of Apoptosis

Testes embedded in paraffin were serially sectioned at 7 µm. Deparaffinized sections were hydrated, microwaved for 5 min in an antigen unmasking solution (Vector Laboratories, Inc., AbCys, Paris, France), and left to cool to room temperature. The sections were washed in a PBS bath for 5 min and immersed in peroxidase blocking reagent at room temperature for 10 min to quench endogenous peroxidase activity (DAKO Cytomation, Dako, Ely, UK). After two PBS baths for 5 min, nonspecific background was prevented by incubation in 5% lamb serum/PBS for 30 min. Finally, the sections were incubated with PBS containing primary antibody against chicken Vasa overnight at 4 °C [46]. The following day, after two PBS baths for 5 min, sections were incubated for 30 min at room temperature with “ready to use” labeled Polymer-HRP anti-rabbit (DAKO Cytomation, Dako, Ely, UK). Finally, after two baths in PBS, staining was revealed by incubation with a 3,3′-diaminobenzidine tablet (Sigma, l’Isle d’Abeau Chesnes, France) dissolved in deionized water at room temperature. Negative controls were incubated with rabbit IgG.

Apoptosis was detected on deparaffinized sections hydrated and permeabilized with Proteinase K as described in the instruction manual (FragEL kit, Calbiochem, VWR, West Chester, PA, USA). Endogenous peroxidases were inactivated by incubation in 3% H_2_O_2_, then the nuclear DNA fragments were end-labeled in situ in a humid chamber for 1.5 h at 37  °C by terminal deoxynucleotidyl transferase (TdT). Staining was revealed with 3,3′-diaminobenzidine (DAB), negative controls were TdT-free [47].

### 4.6. Liver Fractions and Protein Concentration

Four grams of liver were homogenized in 12 mL pH 7.4 phosphate buffer (0.1 M, 0.1 M Tris acetate, 0.1 M KCl, 1 mM EDTA and 0.02 M butylated hydroxytoluene) at 4 °C. The homogenate was centrifuged at 9000× *g*, for 30 min at 4 °C, and the supernatant (S9) was collected. Five hundred microliters of the S9 fraction were deproteinized with metaphosphoric acid (1.25 M, *vol*/*vol*) and stored at −80 °C until further analysis to determine glutathione content. The remainder of the S9 fraction was stored at −80 °C for further analysis. The protein concentration in the S9 fraction was measured using the Bio-Rad kit protein assay (Bio-Rad Laboratories, München, Germany).

### 4.7. Oxidative Markers and Antioxidant Enzyme Activity in Plasma and Liver

Malondialdehyde (MDA) was measured fluorometrically (excitation: 515 nm, emission: 548 nm) in plasma and in the S9 fraction of liver (1 mg protein/mL) after butanol extraction of a pink complex formed with thiobarbituric acid [48,49,50]. Total glutathione (TGlu) was measured with Ellman’s reagent using an optimized enzymatic recycling method for the quantification of reduced glutathione (GSH). Briefly, GSH reacts with 5,5′-dithio-*bis*-(2-nitrobenzoic acid) to produce 5-thio-2-nitrobenzoic acid (TNB) and Gs-TNB. Both oxidized glutathione (GSSG) and Gs-TNB are reduced by glutathione reductase to recycle the GSH, which produces more TNB. The rate of TNB was measured spectrophotometrically at 405 nm. Dilution (1/100) of the deproteinized S9 fraction was required before it was measured in the S9 samples. Oxidized glutathione, exclusive of GSH, was obtained by the first derivatization of GSH with 1 M 2-vinylpyridine before measurement of total glutathione (Baker et al., 1990; Griffith, 1980).

Superoxide dismutase (SOD, EC 1.15.1.1) activity was measured in plasma (5 mg protein/mL) and in the S9 fraction of liver (50 µg protein/mL). Measurement was based on the inhibition of the formation of blue formazan by xanthine-xanthine oxidase (EC 1.17.3.2) in the presence of nitroblue tetrazolium (Sun et al., 1988). The reaction was monitored spectrophotometrically at 540 nm, and SOD activity was calculated by linear regression using bovine erythrocyte SOD as standard. Catalase (CAT, EC 1.11.1.6) activity was estimated by the formation of formaldehyde from methanol in the presence of H_2_O_2_. Formaldehyde reacts with Purpald reagent to form a complex that is dosed at 540 nm (Johansson and Håkan Borg, 1988). Glutathione reductase (GR, EC 1.6.4.2) reduces GSSG into GSH in the presence of nicotinamide adenosine phosphate (NADPH) (Carlberg and Mannervik, 1984). Glutathione peroxidase (GPx, EC 1.11.1.9) reduces cumene hydroperoxide in the presence of GSH to produce GSSG, which is recycled into GSH by GR in the presence of NADPH (Paglia and Valentine, 1967). GR and GPx activities were measured separately in plasma (5 mg protein/mL, 12 min of kinetics) and in the S9 fraction (1 mg protein/mL, 2 min of kinetics) by monitoring the decrease in NADPH content at 340 nm.

### 4.8. Testis Extracts and Assays

Testis extracts were obtained after three repeated freeze/thaw cycles in PBS. The protein concentration in the supernatant was determined using a colorimetric assay kit (Uptima Interchim, Montluçon, France).

Caspase 3/7 Glow assays were performed according to the manufacturer’s instructions (Promega, Charbonnieres les Bains, France). The quantification of caspase-3/7 activity uses a luminogenic substrate containing the sequence asp-glu-val-asp, and after cleavage by caspases, a luciferin substrate is liberated and is used by luciferase to generate light. The amount of luminescence is proportional to the amount of caspase activity present in the cell lysate. Luminescence was measured in relative light units and normalized to 100,000 seminiferous tubule cells.

The chicken proliferating cell nuclear antigen (PCNA) was measured to analyze cell proliferation, whereas when azoospermia-like protein (DAZL) is deleted, this is a marker of spermatogonia used to reveal the immature germ cell content. Interleukin 1β (Ilβ), and interferon-γ (IFN-γ) concentrations were quantified to assess inflammatory response in the testis. All standards and samples were assayed according to the manufacturer’s recommendations (Cusabio, Eurobio, Courtaboeuf, France).

Total antioxidant capacity (TAC) and catalase activity were measured by the Cayman’s antioxidant assay kit according to the instruction manual (Cayman, Interchim, Montluçon, France). Assay plates were red by using an ELISA plate reader at 750 nm for TAC and at 540 nm for catalase activity. The TAC assay is based on the ability of antioxidants (vitamins, proteins, lipids, glutathione, uric acid, etc.) to inhibit the oxidation of 2,2′-azino-bis,3-ethylbenzthiazoline-6-sulphonic acid. Total antioxidant capacity is expressed as Trolox equivalent antioxidant capacity per mg of testicular protein extract.

The concentration of cAMP was measured using the cAMP-Glo assay as recommended by the manufacturer (Promega, Madison, WI, USA). The concentration of testosterone was assessed by radioimmunoassay in duplicate as previously described [51]. The sensitivity test was 15 pg/tube and intra-assay coefficients of variation of 5.3%.

### 4.9. Determination of the Sphinganine to Sphingosine Ratio

Calculation of the sphinganine (Sa) to sphingosine (So) ratio requires prior determination of free Sa and free So contents (Riley et al., 1994a). Briefly, 0.2 nmol of C_20_ sphinganine (Matreya, Inc., Pleasant Gap, PA, USA) used as internal standard were added to 100 µL of plasma or S9. Sphingolipids were extracted by alkaline methanolic-chloroform and the chloroform phase was washed twice with alkaline water. Extracts were dried under nitrogen flow, then suspended in 20 µL methanol, and sonicated for 10 min. Derivatization of sphingolipid was performed with ortho-phthalaldehyde before injection using automate (ICS M2200, Toulouse, France). The HPLC system is composed of an ICS M2200 HPLC autosampler (ICS, Toulouse, France) that delivers methanol–water (90:10, *v*/*v*) to a Prontosil C18 cartridge equipped with a C18 pre-column filter (Bischoff, Leonberg, Germany). A fluorescence detector (FD-500 Shimazu, Kyoto, Japan) was used for detection, the excitation wavelength was 335 nm, and the emission wavelength 440 nm. Mean retention times were 12, 17 and 29 min for sphingosine, sphinganine and C_20_ sphinganine, respectively. Concentrations were calculated by linear regression from standard solutions that were injected daily. C_20_ sphinganine was used as an internal standard to monitor the extraction rate the sphingolipids in the samples.

### 4.10. Statistical Analysis

Data on all response variables are reported as means ± SD or SEM and were the subject of one-way analysis of variance (ANOVA) after a normality test (Shapiro-Wilk). When a significant difference was observed (*p* < 0.05), the difference between means was determined by individual comparison of means (Kruskall–Wallis). Statistically different groups (*p* < 0.05) are identified by a different letter. All statistical analyses were conducted by XLSTAT Biomed.

## Figures and Tables

**Table 1:** Mycotoxin levels in the experimental diets fed to broiler chickens from 0 to 35 days of age.

Mycotoxins	Experimental Diets				
	Control ^1^	DON ^1^	FB ^1^	ZON ^1^	DONFBZON ^1^
Deoxynivalenol					
Expected	0	5000	0	0	5000
Measured 0–10	50	4570	60	55	3820
Measured 11–35	50	4720	65	50	4170
Fumonisins					
Expected ^2^	0	0	20,000	0	20,000
Measured 0–10 ^3^	25/<10/<10	30/<10/<10	19,500/1600/2000	70/10/15	17,600/1440/2050
Measured 11–35 ^3^	35/<10/<10	30/<10/<10	21,000/2130/2300	90/15/20	17,700/1530/2030
Zearalenone					
Expected	0	0	0	500	500
Measured 0–10	35	40	35	465	415
Measured 11–35	25	25	25	480	430

^1^ As described in Material and Methods, 10 experimental diets were formulated to provide respective total protein and metabolizable energy of 22% and 2880 kcal/kg for chickens from 0 to 10 days of age and 19.5% and 3050 kcal/kg for chickens from 11 to 35 days of age. Expected and measured concentrations of fusariotoxins (µg/kg) in the diets contaminated by deoxynivalenol alone (DON), fumonisins alone (FB), zearalenone alone (ZON) and a mixture of the three toxins (DONFBZON). ^2^ Sum of FB1 + FB2. ^3^ Concentrations of fumonisin B1 (FB1), fumonisin B2 (FB2) and fumonisin B3 (FB3), respectively.

**Table 2:** Effects of fusariotoxins on performances and relative organ weights in broiler chickens ^1^.

Variable ^3^	Control ^2^	DON ^2^	FB ^2^	ZON ^2^	DONFBZON ^2^
BW (D11)	257 ± 35	248 ± 31	265 ± 37	263 ± 34	273 ± 24
BW (D35)	2248 ± 134	2235 ± 19	2358 ± 249	2395 ± 214	2404 ± 143
FC (0–10)	248 ± 37	247 ± 37	265 ± 42	265 ± 39	271 ± 27
FC (11–35) *	3058 ± 193 ^a^	3038 ± 236 ^a^	3232 ± 299 ^a,b^	3303 ± 271 ^b^	3289 ± 180 ^b^
DWG (0–10)	19.2 ± 3.3	18.5 ± 2.8	20 ± 3.4	19.8 ± 3.2	20.7 ± 2.1
DWG (11–35)	83 ± 4.8	82.8 ± 7	87.2 ± 9.3	88.8 ± 7.9	88.8 ± 5.5
FCR (0–10)	1.18 ± 0.05	1.22 ± 0.05	1.21 ± 0.05	1.22 ± 0.06	1.19 ± 0.03
FCR (11–35)	1.54 ± 0.03	1.53 ± 0.03	1.55 ± 0.04	1.55 ± 0.04	1.54 ± 0.04
Liver RW *	1.99 ± 0.19 ^a,b^	1.87 ± 0.19 ^b^	1.92 ± 0.16 ^b^	2.09 ± 0.18 ^a^	1.9 ± 0.12 ^b^
Kidney RW	0.61 ± 0.06	0.62 ± 0.04	0.62 ± 0.06	0.6 ± 0.05	0.62 ± 0.06
Intestine RW	2.1 ± 0.40	2.1 ± 0.25	2.11 ± 0.2	2.06 ± 0.23	2.01 ± 0.22
Caecum RW	0.27 ± 0.04	0.26 ± 0.05	0.28 ± 0.05	0.26 ± 0.04	0.26 ± 0.04
Gizzard RW	1.07 ± 0.23	1.1 ± 0.17	1.22 ± 0.26	1.05 ± 0.12	1.05 ± 0.22
Hearth RW	0.69 ± 0.08	0.71 ± 0.07	0.64 ± 0.07	0.7 ± 0.06	0.69 ± 0.08
Tonsil RW	0.06 ± 0.01	0.05 ± 0.01	0.06 ± 0.01	0.05 ± 0.01	0.06 ± 0.01
Bursa RW	0.22 ± 0.03	0.22 ± 0.05	0.23 ± 0.06	0.20 ± 0.03	0.19 ± 0.05

^1^ Values were obtained from 14 animals per group and are expressed as mean ± SD. ANOVA was performed to compare groups. When a significant difference was observed (* *p* < 0.05), means were compared (Kruskall–Wallis). Different letters in the same row identify statistically different groups (*p* < 0.05). ^2^ Experimental diets were formulated as described in Material and Methods to reach a final concentration of 5 mg DON/kg diet (DON), 20 mg FB1 + FB2/kg diet (FB), 0.5 mg ZON/kg diet and 5 mg DON + 20 mg FB1 + FB2 + 0.5 mg ZON/kg diet (DONFBZON). The control diet was free of mycotoxins. ^3^ Results are expressed in the following units: Feed consumption (FC), g; daily weight gain (DWG), g; feed conversion ratio (FCR) calculated from D0 to D10 and from D11 to D35. Relative organs weights (RW) measured on D35 after killing.

**Table 3:** Effects of fusariotoxins on the density and morphometry of segments of the small intestinal in broiler chickens ^1^.

Variable ^3^	Control ^2^	DON ^2^	FB ^2^	ZON ^2^	DONFBZON ^2^
Density					
Duodenum	0.35 ± 0.03	0.36 ± 0.04	0.37 ± 0.04	0.38 ± 0.05	0.36 ± 0.05
Jejunum	0.32 ± 0.04	0.31 ± 0.03	0.31 ± 0.03	0.32 ± 0.06	0.33 ± 0.05
Ileum	0.24 ± 0.03	0.22 ± 0.03	0.24 ± 0.04	0.23 ± 0.05	0.23 ± 0.04
Duodenum					
Villus height	1.28 ± 0.14	1.29 ± 0.13	1.29 ± 0.14	1.43 ± 0.2	1.3 ± 0.14
Villus width	684 ± 67	625 ± 82	708 ± 127	683 ± 149	658 ± 103
Crypt depth	141 ± 12	138 ± 12	142 ± 12	147 ± 9	143 ± 12
Crypt width	47 ± 3	45 ± 3	46 ± 3	46 ± 4	44 ± 4
Goblet cells	107 ± 18	123 ± 18	110 ± 18	113 ± 17	123 ± 90
Jejunum					
Villus height	1.15 ± 0.12	1.2 ± 0.16	1.22 ± 0.11	1.14 ± 0.15	1.16 ± 0.11
Villus width	659 ± 134	658 ± 151	676 ± 126	738 ± 159	679 ± 62
Villus area	756 ± 151	801 ± 236	831 ± 192	838 ± 208	786 ± 108
Crypt depth *	99 ± 7 ^a^	130 ± 18 ^b^	145 ± 14 ^c^	145 ± 19 ^c^	152 ± 12 ^c^
Crypt width	40 ± 3	44 ± 3	41 ± 5	42 ± 4	44 ± 4
Crypt area *	4 ± 1 ^a^	6 ± 1 ^b^	6 ± 1 ^b^	6 ± 1 ^b^	7 ± 1 ^b^
Villus/Crypt *	11.65 ± 0.82 ^a^	9.25 ± 1.28 ^b^	8.46 ± 0.82 ^b,c^	7.84 ± 1.03 ^c^	7.64 ± 0.60 ^c^
Goblet cells	128 ± 23	133 ± 24	131 ± 23	127 ± 27	141 ± 22
Ileum					
Villus height	0.787 ± 0.094	0.847 ± 0.089	0.831 ± 0.119	0.808 ± 0.157	0.903 ± 0.181
Villus width	428 ± 44	402 ± 43	407 ± 71	438 ± 67	459 ± 114
Crypt depth	141 ± 13	139 ± 11	139 ± 16	140 ± 14	145 ± 15
Crypt width	44 ± 4	45 ± 3	45 ± 3	43 ± 4	45 ± 2
Goblet cells	193 ± 22	192 ± 26	186 ± 34	182 ± 38	194 ± 35

^1^ Values were obtained from 14 animals per group and are expressed as mean ± SD. ANOVA was performed to compare groups. When a significant difference was observed (* *p* < 0.05), means were compared (Kruskall–Wallis). Different letters in the same row identify statistically different groups (*p* < 0.05). ^2^ Experimental diets were formulated as described in Material and Methods to reach a final concentration of 5 mg DON/kg diet (DON), 20 mg FB1 + FB2/kg diet (FB), 0.5 mg ZON/kg diet and 5 mg DON + 20 mg FB1 + FB2 + 0.5 mg ZON/kg diet (DONFBZON). The control diet was free of mycotoxins. ^3^ Results are expressed in the following units: Villus height, mm; villus width, crypt depth, crypt width, µm; villus surface area, crypt surface area, mm^2^×1000; goblet cells, number of cells/0.02 mm^2^.

**Table 4:** Effects of fusariotoxins on biochemistry and hematology in broiler chickens ^1^.

Variable ^3^	Control ^2^	DON ^2^	FB ^2^	ZON ^2^	DONFBZON ^2^
Proteins	30.9 ± 4.5	30.5 ± 2.5	30.9 ± 2.9	32.4 ± 9	31.6 ± 4.3
Cholesterol	1.51 ± 0.15	1.55 ± 0.19	1.5 ± 0.12	1.51 ± 0.21	1.45 ± 0.11
Uric acid *	40.9 ± 13.2 ^a^	40.1 ± 9.2 ^a^	47.1 ± 18.3 ^a,b^	59.9 ± 16.3 ^b^	50.5 ± 11.6 ^a,b^
LDH	3450 ± 952	2779 ± 762	3241 ± 690	3598 ± 1635	3110 ± 842
ALP	2133 ± 925	1706 ± 499	2049 ± 816	2,180 ± 953	1664 ± 449
ALT	5.71 ± 3.5	5.71 ± 3.2	6.29 ± 4.2	4.43 ± 3.44	5.21 ± 3.51
IgA	221 ± 54	176 ± 73	171 ± 49	163 ± 52	194 ± 59
Hb	13.5 ± 1.1	13.7 ± 0.7	13.7 ± 0.9	13.3 ± 1.2	13.7 ± 1.1
Erythrocytes	2.42 ± 0.21	2.51 ± 0.2	2.47 ± 0.26	2.4 ± 0.25	2.51 ± 0.22
Leucocytes	20.3 ± 2.2	19.5 ± 1.4	19.2 ± 1.4	19.4 ± 1.9	20 ± 1.7

^1^ Values were obtained from 14 animals per group aged 35 days and are expressed as mean ± SD. One-way ANOVA was performed to compare groups. When a significant difference was observed (* *p* < 0.05), means were compared (Kruskall–Wallis). Different letters in the same row identify statistically different groups (*p* < 0.05). ^2^ Experimental diets were formulated as described in Materials and Methods to reach a final concentration of 5 mg DON/kg diet (DON), 20 mg FB1 + FB2/kg diet (FB), 0.5 mg ZON/kg diet and 5 mg DON + 20 mg FB1 + FB2 + 0.5 mg ZON/kg diet (DONFBZON). The control diet was free of mycotoxins. ^3^ Results are expressed in the following units: Proteins, g/L; cholesterol, mmol/L, uric acid, mmol/L; lactate dehydrogenase (LDH), UI/L; alkaline phosphatase (ALP), UI/L; alanine aminotransferase (ALT), UI/L; immunoglobulin A(IgA), µg/L), hemoglobin (Hb), mg/L; erythrocytes and leucocytes, 10^3^ number of cells/mL.

**Table 5:** Effects of fusariotoxins on oxidative stress variables in broiler chickens ^1^.

Variable ^3^	Control ^2^	DON ^2^	FB ^2^	ZON ^2^	DONFBZON ^2^
Plasma					
MDA	683 ± 184	734 ± 233	676 ± 174	600 ± 213	608 ± 220
TGs	7.8 ± 1.4	8.1 ± 2.6	6.9 ± 1.8	6.8 ± 2.2	6.6 ± 1.7
SOD	136 ± 28	128 ± 47	136 ± 52	134 ± 63	109 ± 52
CAT	5.5 ± 1.3	5.4 ± 1.2	4.8 ± 0.5	5.2 ± 0.8	5.1 ± 1.1
GsPx	144 ± 44	132 ± 22	138 ± 20	132 ± 19	137 ± 26
GsRed	7.9 ± 3.6	9.3 ± 3.1	10.1 ± 3.2	8.8 ± 3	8.8 ± 3.3
Liver					
MDA	13.3 ± 5.7	11 ± 4.8	11.5 ± 6.4	11.7 ± 5.1	13.2 ± 6
TGs	22.3 ± 5.5	24.8 ± 8.4	24.6 ± 6.1	24.8 ± 8.1	21.5 ± 5.7
GSSG	3.8 ± 1.2	4.7 ± 2.2	4.2 ± 2.2	4.9 ± 2.2	3.4 ± 1.3
SOD	20.4 ± 5.3	18.1 ± 8.1	18.5 ± 6.7	18.3 ± 8.3	18.6 ± 4.6
CAT	16.6 ± 5.4	20 ± 5.6	20.9 ± 9.6	17.1 ± 8	16.7 ± 6.3
GPx	37.9 ± 8.7	39.2 ± 3.6	39.4 ± 7.5	39 ± 4.9	37.9 ± 4.1
GRed	14 ± 2.2	15.3 ± 2.1	15.3 ± 3.8	13.2 ± 2.6	14.1 ± 3.3

^1^ Values were obtained from 14 animals per group and are expressed as mean ± SD. One-way ANOVA was performed to compare groups. No significant difference among groups was observed (*p* > 0.05). ^2^ Five experimental diets were formulated as described in Material and Methods to reach a final concentration of 5 mg DON/kg diet (DON), 20 mg FB1 + FB2/kg diet (FB), 0.5 mg ZON/kg diet and 5 mg DON + 20 mg FB1 + FB2 + 0.5 mg ZON/kg diet (DONFBZON). The control diet was free of mycotoxins. ^3^ Results are expressed per L of plasma or per mg of S9 liver proteins in the following units: Malondialdehyde (MDA), nmol; total glutathione (TGs), µmol GSSG; oxidized glutathione (GSSG), µmol; superoxide dismutase (SOD), U/min; catalase (CAT), µg of formaldehyde (CH_2_O)/min; glutathione peroxidase (GPx), µmol nicotinamide adenine dinucleotide phosphate (NADPH)/min; glutathione reductase (GRed), µmol NADPH/min.

**Table 6:** Effects of fusariotoxins on testes in broiler chickens ^1^.

Variable ^3^	Control ^2^	DON ^2^	FB ^2^	ZON ^2^	DONFBZON ^2^
Testis	207 ± 14	244 ± 25	148 ± 17	263 ± 21	230 ± 16
STD	56.5 ± 1.6	54.7 ± 1	52.1 ± 0.7	66.5 ± 1.5	44.8 ± 0.7
Cleaved caspase ^3^	4.33 ± 0.46	4.16 ± 0.68	2.48 ± 0.36	3.12 ± 0.47	3.52 ± 0.68
DAZL *	0.28 ± 0.02 ^a^	2.24 ± 0.96 ^b^	8.18 ± 2.78 ^c^	1.02 ± 0.24 ^b^	0.89 ± 0.42 ^b^
PCNA *	1.19 ± 0.11 ^a^	2.49 ± 0.45 ^a^	4.66 ± 0.70 ^b^	2.13 ± 0.55 ^a^	1.57 ± 0.33 ^a^
IFN-γ	0.58 ± 0.12	0.92 ± 0.07	0.49 ± 0.16	0.46 ± 0.12	0.25 ± 0.04
Il1β	4.59 ± 2.70	6.71 ± 3.50	4.84 ± 2.18	3.18 ± 1.69	2.23 ± 0.57
TAC	7.30 ± 1.90	9.95 ± 2.76	9.63 ± 1.71	14.51 ± 3.88	9.61 ± 1.33
CAT	14.68 ± 4.50	17.34 ± 4.91	12.79 ± 0.87	11.63 ± 1.73	5.55 ± 1.02
cAMP	219 ± 48	594 ± 224	1447 ± 335	1094 ± 325	1321 ± 385
Testosterone	396 ± 199	288 ± 105	443 ± 150	402 ± 156	448 ± 177

^1^ Values are expressed as mean ± SEM. One-way ANOVA was performed to compare groups. When a significant difference was observed (* *p* < 0.05), means were compared (Kruskall–Wallis). Different letters in the same row identify statistically different groups (*p* < 0.05). ^2^ Experimental diets were formulated as described in Material and Methods to reach a final concentration of 5 mg DON/kg diet (DON). 20 mg FB1 + FB2/kg diet (FB). 0.5 mg ZON/kg diet and 5 mg DON + 20 mg FB1 + FB2 + 0.5 mg ZON/kg diet (DONFBZON). The control diet was free of mycotoxins. ^3^ Results are expressed in the following units: Testis weight, mg; seminiferous tubules diameter, µm; caspase 3 activity, relative luminescence units (RLU)/mg protein; deleted in azoospermia-like (DAZL), ng/mg protein; proliferating cell nuclear antigen (PCNA), ng/mg protein; interferon gamma (IFN-γ), pg/mg protein; interleukin 1 beta (Il1β), pg/µg proteins; total antioxidant capacity (TAC), trolox equivalent antioxidant capacity/mg proteins; catalase (CAT) activity, in pmol/min/mL/µg proteins; cAMP, µMolar/mg proteins; testosterone, ng/µg proteins.

## Abbreviations

FUS	fusariotoxins
FB_1_	fumonisin B1
FB_2_	fumonisin B2
FB_3_	fumonisin B3
DON	deoxynivalenol
ZON	zearalenone
BW	body weight
DWG	daily weight gain
FCR	feed conversion ratio
RW	relative weights
LDH	lactate dehydrogenase
ALP	alkaline phosphatase
ALT	alanine aminotransferase
IgA	Immunoglobulin A
Hb	hemoglobin
MDA	malondialdehyde
TGlu	total glutathione
GSSG	oxidized glutathione
GSH	reduced glutathione
SOD	superoxide dismutase
CAT	catalase
CH_2_O	formaldehyde
GPx	glutathione peroxidase
GRed	glutathione reductase
NADPH	nicotinamide adenine dinucleotide phosphate
HES	hematoxylin; eosin and saffron
TdT	deoxynucleotidyl transferase
DAB	3,3′-diaminobenzidine
TAC	Total antioxidant capacity
Ilβ	Interleukin 1β
IFN-γ	Interferon-γ
PCNA	proliferating cell nuclear antigen
DAZL	deleted in azoospermia-like
Sa	sphinganine
So	sphingosine
